# Investigating coaches’ general beliefs on defining and identifying talent in basketball

**DOI:** 10.3389/fpsyg.2025.1701313

**Published:** 2025-11-21

**Authors:** Till Koopmann, Arne Chorengel, Jörg Schorer

**Affiliations:** Sport and Movement, Institute of Sport Science, Carl von Ossietzky Universität Oldenburg, Oldenburg, Germany

**Keywords:** athlete development, athlete identification, athlete selection, talent development, talent identification, talent selection, youth sports, coach’s eye

## Abstract

Coaches have various challenging tasks to handle, including athlete development and selection. Selection decisions are often based on the coach’s eye. That is, coaches use their experience and intuition to generate holistic and subjective evaluations of athletes as the basis for selection decisions. In this context, both general beliefs regarding talent (in sports) and sport-specific aspects play an important role. Research investigating coaches’ thought and decision-making processes underlying selection decisions is rare. Thus, the present study applied an online questionnaire with *n* = 125 basketball coaches to investigate coaches’ general beliefs regarding talent (in basketball). Findings show four themes. First, coaches believe talent (in basketball) is identifiable, multidimensional, and compensatory. That is, it is defined by the interplay of multiple characteristics that can compensate for each other within a holistic athlete profile. Second, coaches reported that not only the importance of players’ characteristics but also their own definition of talent (in basketball) change over time. Third, coaches show that both individual (e.g., anthropometry, technical skills) and environmental (e.g., family, training) factors are important for player selection. Fourth, coaches appear to include both objectively (e.g., lab-based tests) and subjectively (e.g., game observations) collected information within their decision-making, mainly relying upon the latter. In summary, this study highlights the continued importance of holistic, flexible, and developmental approaches to athlete evaluation. These findings should be included in reflective and evidence-informed coach development systems as coaches appear to remain the main decision-makers selecting athletes based on their coach’s eye.

## 1 Introduction

Coaches must juggle various different tasks and responsibilities. Among others, athlete development comprises the crucial task of evaluating (young) athletes regarding their multidimensional characteristics (e.g., cognitive, physical, psychological; Baker et al., 2019). These evaluations can be used for, e.g., individual training adjustments as well as athlete selection decisions. Previous research has found and argued that the evaluation of athletes is conducted best based on a combination of both objective assessment data (e.g., motor performance tests) and subjective coach observation information (e.g., Sieghartsleitner et al., 2019; Höner et al., 2021).

On the one hand, objective assessments can be more or less complex and representative. This can range from data being acquired during complex competition to isolated assessments in a lab context (Koopmann et al., 2020; Den Hartigh et al., 2018; Pinder et al., 2013). For example, studies can analyse competition data (e.g., points made, assists, rebounds) or measure classic anthropometrics or physiological abilities in isolated tests (e.g., body height and weight, wing span, vertical jump test). On the other hand, subjective coach evaluations are often relying on the so-called *coach’s eye* used during practice or competition observations (Lath et al., 2021; Roberts, 2021). The coach’s eye is defined as being holistic, intuitive, subjective and experience-based, and with that is discussed similarly to coaches’ intuitive judgement and *gut instinct* (Roberts et al., 2021). For basketball, the sport’s highly complex nature has led to coach’s eye-based practice and game observations being the central evaluation method that is enriched with more and more objective data (Rogers et al., 2022).

One rather open question is the best way *how to acquire which* information and *how to combine* it for different (sport) contexts. Subjective coach evaluations as human thought and decision-making processes are highly complex and can be influenced by various biases, e.g., relative age effects or confirmation bias (Johnston and Baker, 2019; Johnston and Baker, 2024). So far, research has used three approaches to investigate the coach’s eye and its underlying mechanisms, aiming to understand and improve evaluations and decisions: (1) Qualitative interviews (Cook et al., 2014; Koopmann et al., 2023), (2) online questionnaires (Larkin et al., 2023; Rogers et al., 2022), and (3) experimental study designs (Lath et al., 2025).

Qualitative interview studies investigated coaches’ beliefs in different sports. For example, coaches from different sports games were interviewed and reported anthropometric and physiological characteristics, game-specific skills and performance, and players’ psychological qualities are important in adolescent players (e.g., Chiwaridzo et al., 2019; Larkin et al., 2022; Cook et al., 2014). Similarly, interview studies on coaches’ beliefs on individual aspects of talent and athlete selection in basketball found that experts coaches have a multidimensional understanding including mainly anthropometrics and athleticism, fundamental motor abilities, psychological factors and tactical and technical skills (Larkin et al., 2023; Koopmann et al., 2025).

Other studies followed the second approach to coach’s eye research and applied online questionnaires with (basketball) coaches. Those studies identified a holistic multidisciplinary approach to talent identification including psychological, tactical decision-making, physical and motor as well as technical skill indicators (Rogers et al., 2022; Larkin et al., 2023). Koopmann et al. (2025) used a combination of interviews and a online questionnaire on the importance of individual aspects in basketball. As exemplary findings from the online questionnaire analysis, coaches showed that motor abilities are important all throughout different age groups ranging from 8–21 years of age while technical skills become more important later on. That is, athlete age has a crucial impact on coaches’ evaluation process and emphasizes the multidimensional and dynamic nature of talent (e.g., Baker et al., 2019). These findings are in accordance with studies not investigating the coach’s eye per se but comparing, e.g., selected and non-selected players in basketball. In those studies, selected players tend to have higher muscular power and be taller in combination with better basketball-specific tactical (i.e., perceptual-cognitive) and technical skills (Guimarães et al., 2019; Joseph et al., 2021; Rösch et al., 2022).

Studies applying the third approach investigating the coach’s eye using experimental study designs are rare. In a recent study, Lath et al. (2025) used the iCodes model (Jekel et al., 2018) to explore coaches’ information search in athlete selection. Results showed evidence for the ideas that (1) coaches want more information regarding aspects they view as more important, and (2) coaches from different sport contexts differ regarding their information search. Findings regarding the attraction search effect (i.e., coaches search for information regarding the more attractive athlete) were indeterminate (Lath et al., 2025).

The larger part of previous studies on athlete selection focused on individual and sport-specific aspects and coaches’ evaluation thereof. Besides these specific aspects and their undeniable importance, also coaches’ basic assumptions and general beliefs regarding talent (in sports in general and in basketball specifically) form the fundament for athlete evaluations. These general beliefs can be investigated based on widespread themes discussed in previous talent research including athlete selection and development in sports (e.g., Baker et al., 2020).

One of those research themes is the idea that the overall evaluation process is influenced by how a coach defines talent and whether a coach believes it is identifiable at all. Based on various definitions of talent in sports, talent may be defined as, for example, multidimensional and dynamic (e.g., Baker et al., 2019). Here, multidimensionality refers to the fact that talent comprises a combination of different individual characteristics (e.g., physical, psychological), and the dynamics emphasize the idea that talent evolves across time. In this context, experts and coaches often believe performance and talent in basketball are characterized by the compensation phenomenon (Vaeyens et al., 2008; Koopmann et al., 2025). Accordingly, players may compensate a weakness or lower skill level in one with a strength or higher skill level in another area (e.g., lower height with extraordinary explosiveness or great technical shooting skills; Larkin et al., 2023; Koopmann et al., 2025). This aspect is also relevant given different positions and roles in various sports and in basketball specifically. Despite tendencies towards playing styles or philosophies including an “positionless basketball” approach favoring versatile all-round players, demands of different positions and roles may still influence coaches’ evaluations.

Another research theme is whether coaches believe that talent identification is done best based on individual or environmental information (e.g., Issurin, 2017). In addition, a third widespread research theme is whether athletes should be evaluated based on subjective or objective data (e.g., Sieghartsleitner et al., 2019). For example, research in different sports has made the important differentiation between coaches evaluating players’ current performance level versus their talents or potential for future performance (Till and Baker, 2020). While these two can be similarly high or low, they can also differ given crucial impacting factors like training age (i.e., experience in the sport) and biological age (i.e., maturation; e.g., Arede et al., 2021). Further understanding coaches’ general beliefs on talent besides sport-specific aspects may improve knowledge and quality of selection decisions.

In summary, some knowledge regarding important aspects for coaches’ player selections and development in basketball exists. However, coaches’ basic assumptions and beliefs and the acceptance of widespread research themes about talent and player evaluation is unclear, especially given crucial factors such as athlete age. This includes the crucial question of how respective data should be acquired and afterwards interpreted leading to (selection) decisions. Thus, the aim of the present study was to investigate basketball coaches’ general beliefs on defining and identifying talent as the basis for understanding the *coach’s eye* in basketball.

## 2 Methods

### 2.1 Participants

A total of 125 basketball coaches participated in this study by filling out the complete online questionnaire. Coaches ranged from the lowest (C-license; *n* = 32) to the highest formal coaching level in Germany (A-license; *n* = 36) and from coaching mainly under-8 teams to coaching professional adult basketball. Coaches showed a wide range of quantitative coaching experience (*M* = 13.4, *SD* = 8.3; *Min* = 1, *Max* = 50 years of experience).

### 2.2 Data collection

All procedures were in full compliance with the Declaration of Helsinki and approved by the ethical committee of the Carl von Ossietzky Universität Oldenburg in Germany (reference: Drs.EK/2024/032). The questionnaire was developed based on the combination of findings from a preparatory qualitative interview study with expert coaches (Koopmann et al., 2025) and previous research in the field. It was created during multiple researcher triangulation and peer debriefing meetings in combination with internal pilot testing. The questionnaire was distributed online in January and February 2024 through authors’ personal networks and basketball clubs with a focus on basketball coaches in Germany from various geographical, performance and experience levels. The questionnaire was created and distributed using the software *LimeSurvey* (LimeSurvey GmbH, Hamburg, Germany). Coaches filled out the questionnaire anonymously. The questionnaire consisted of 21 items across two parts: (1) General understanding of talent evaluation and athlete selection in basketball, and (2) Evaluation of specific individual aspects for different age groups. The present study focuses on the first part of the questionnaire while also presenting one aspect of connecting findings from the second part. A detailed analysis of findings for the specific individual aspects is presented elsewhere (Koopmann et al., 2025). Participating coaches took M = 8.8, SD = 7.4 min to fill out the online questionnaire.

#### 2.2.1 Part 1: general understanding of talent in basketball

Coaches were asked to rate 13 items within four overarching, general themes regarding talent in sport on a scale ranging from 1 (“Strongly Disagree”) to 5 (“Strongly Agree”). Those four research themes (A-D) included the following 13 item statements:

Theme A: “Talent is identifiable, multidimensional and compensatory,” including items (1) “I can identify an athlete’s talent”; (2) “When evaluating players’ talent, I use the same qualities for every player (playing the same position)”; (3) “The qualities integrated in a talent evaluation are differently meaningful”; and (4) “Within my evaluation, one weak quality can be compensated for by a strong quality (e.g., good shooting technique – missing athleticism)”.

Theme B: “Talent and its evaluation are dynamic,” including items (5) “The current performance level plays only an inferior role in my talent evaluation”; (6) “Talent in basketball is referring to a predicted peak performances in adulthood”; (7) “I need multiple evaluation points in time for my talent evaluation”; and (8) “The qualities integrated in my talent evaluation have changed in recent years”.

Theme C: “Individual and environmental factors of talent,” including items (9) “Individual qualities (e.g., technique, personality, anthropometrics) are important for my talent evaluation”; (10) “Environmental factors (e.g., training conditions, parents, social environment) are important for my talent evaluation”; and (11) “The development of a talent’s predicted peak performance requires optimal training conditions (esp. coaches, infrastructure)”.

Theme D: “Objective and subjective parameters of talent,” including items (12) “I need as many objective parameters (e.g., performance test results, questionnaires, statistics) as possible for my talent evaluation,” and (13) “My coach’s eye (esp. intuition, experience) is decisive for my talent evaluation”.

#### 2.2.2 Part 2: age group differentiation

The second part aimed to incorporate the dynamically changing nature of athlete and talent evaluation by differentiating aspects for different age groups. Coaches were asked to rate items regarding the importance of specific aspects from 1 (“Less important”) to 5 (“Very important”) for the three different age groups 8–11, 12–16 and 17–21 years of age. These age groups were chosen based on the German basketball development system consisting of (a) basic youth sport, (b) advanced level organized by federal states including the national under-16 league, and (c) transition phase into the adult and professional level including the national under-19 league. Items included in the questionnaire covered several *individual aspects* as well as *environmental factors* and players’ *current performance level*, *future performance potential*, and *maturation*. To also include an overall value for individual aspects in the analysis, the score for the individual aspects was calculated as the mean of six aspects: technical skills, athleticism, psychological factors, tactical skills, anthropometrics and motor abilities (for details see Koopmann et al., 2025).

### 2.3 Data analysis

Participants’ ratings were analysed descriptively and for part 2 by means of inferential statistics. The latter included repeated-measures analyses of variance (RM-ANOVA) checking for differences between coaches’ ratings for the different player age groups (8–11, 12–16, 17–21 years of age) using contrasts 8–11 vs. 12–16 and 12–16 vs. 17–21 (only part 2). For this purpose, all importance ratings were assumed to be quasi-metric. Statistical significance levels were set at *α* = 0.05 and effect sizes were calculated where appropriate.

## 3 Results

In the following, results are presented following the questionnaire structure. The first part comprises the coaches’ general understanding of talent evaluation and athlete selection in basketball. The second part presents the results regarding the evaluation of specific aspects for different age groups.

### 3.1 Part 1: general beliefs of talent in basketball

**Figure 1:**
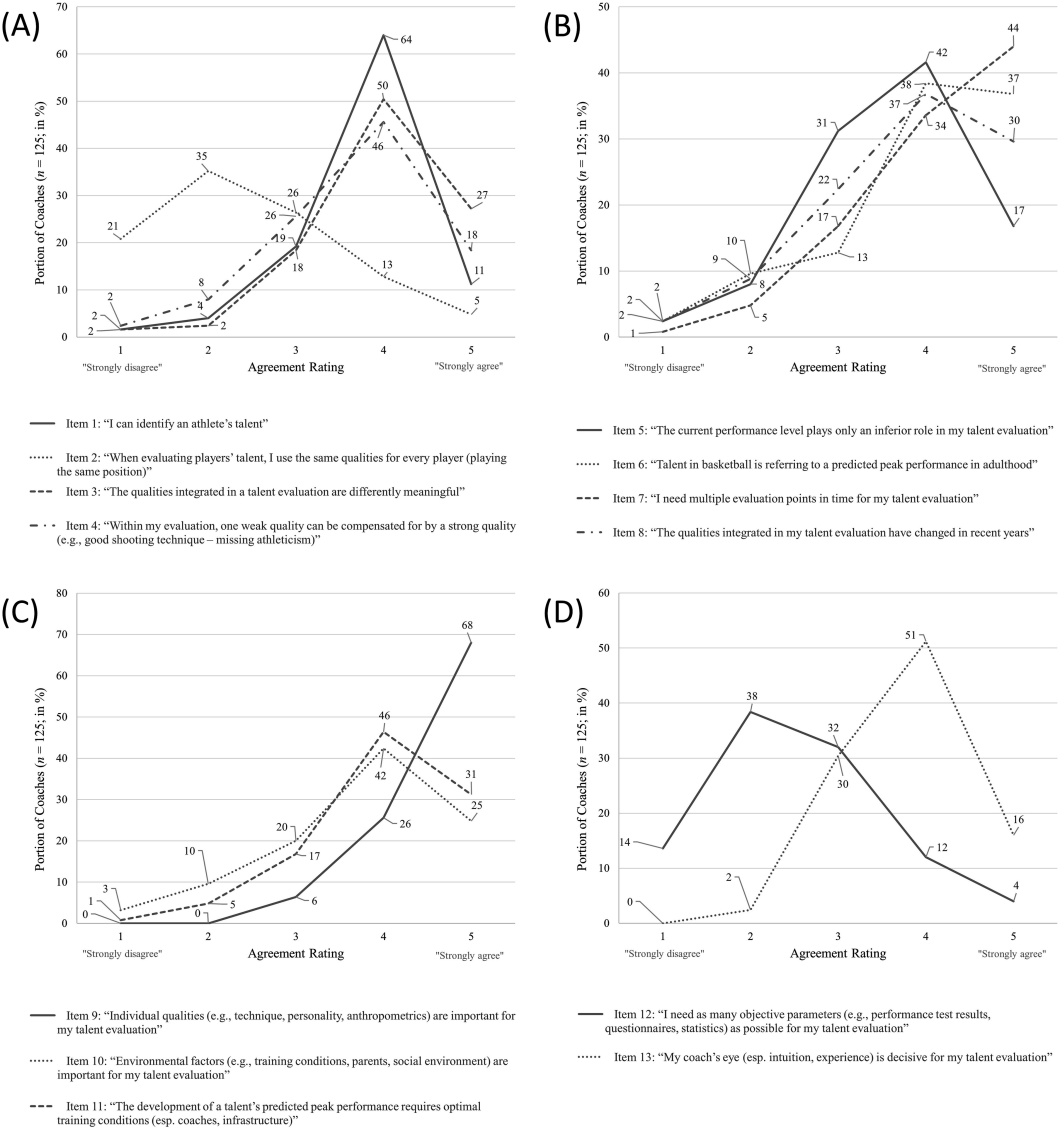
Coaches’ agreement ratings for items concerning themes **(A–D)**; **(A)** “Talent is identifiable, multidimensional, dynamic and compensatory”; **(B)** “Talent and its evaluation are dynamic”; **(C)** “Individual and environmental factors of talent”; **(D)** “Objective and subjective parameters of talent”.

Figure 1 shows coaches’ agreement ratings for all 13 items differentiated by the four research themes A-D.

Coaches showed a more or less strong agreement with most items (overall, most ratings for rating score 4), only items 2 and 12 showed a tendency towards “Strongly disagree” (rating score 1). That is, most coaches feel able to generally identify talent (item 1; see Figure 1A) and appear to do so based on multiple, differently important dimensions/qualities (items 2 and 3) that can interact in compensating ways (item 4).

Furthermore, coaches appear to base their evaluation rather on the future performance potential than the current performance level (items 5 and 6; see Figure 1B) and need multiple observation occasions (i.e., longitudinal data; item 7). Also, coaches showed that they do adjust and change the qualities they include in their evaluation processes (item 8).

The different dimensions/qualities include both individual qualities and environmental factors with even higher agreement ratings for the former (items 9 and 10; see Figure 1C). The environmental factors including training conditions are then considered requirements for the development of peak performance (item 11).

Lastly, coaches show a clear tendency towards their subjective coach’s eye observations and evaluations versus objective data points from tests (items 12 and 13; see Figure 1D).

### 3.2 Part 2: age group differentiation

Table 1 shows the importance ratings for variables *individual aspects (mean)*, *environmental factors*, *current performance level*, *future performance potential*, and *maturation* differentiated for the three age groups. Given statistically significant Mauchly tests, the *F*-values for all analyses were Grennhouse-Geisser adjusted.

**Table 1:** Coaches’ importance ratings for variables differentiated by age groups, and inferential results from RM-ANOVA.

Variables	Importance ratings			Inferential results			
	8–11 years	12–16 years	17–21 years	*F*	*p*	*η_p_^2^* [90% CI]	Power (1-*β*)
Individual aspects (mean)	3.45 (1.06)	4.03 (0.79)	4.47 (0.70)	*F*(1.81, 224.75) = 326.52	<0.001	0.73 [0.68–0.76]	
Environmental factors	3.68 (1.02)	3.96 (0.93)	4.03 (0.92)	*F*(1.87, 231.83) = 9.35	<0.001	0.07 [0.02–0.12]	
Current performance level	2.22 (1.04)	3.25 (0.81)	4.02 (0.84)	*F*(1.78, 221.00) = 207.89	<0.001	0.63 [0.58–0.68]	
Future performance potential	3.79 (1.00)	4.06 (0.83)	4.16 (0.79)	*F*(1.61, 200.09) = 9.61	<0.001	0.07 [0.02–0.13]	
Maturation	3.41 (1.25)	3.58 (1.09)	3.30 (1.30)	*F*(1.38, 171.03) = 2.53	0.102	0.02 [0.00–0.06]	1.00

**Figure 2:**
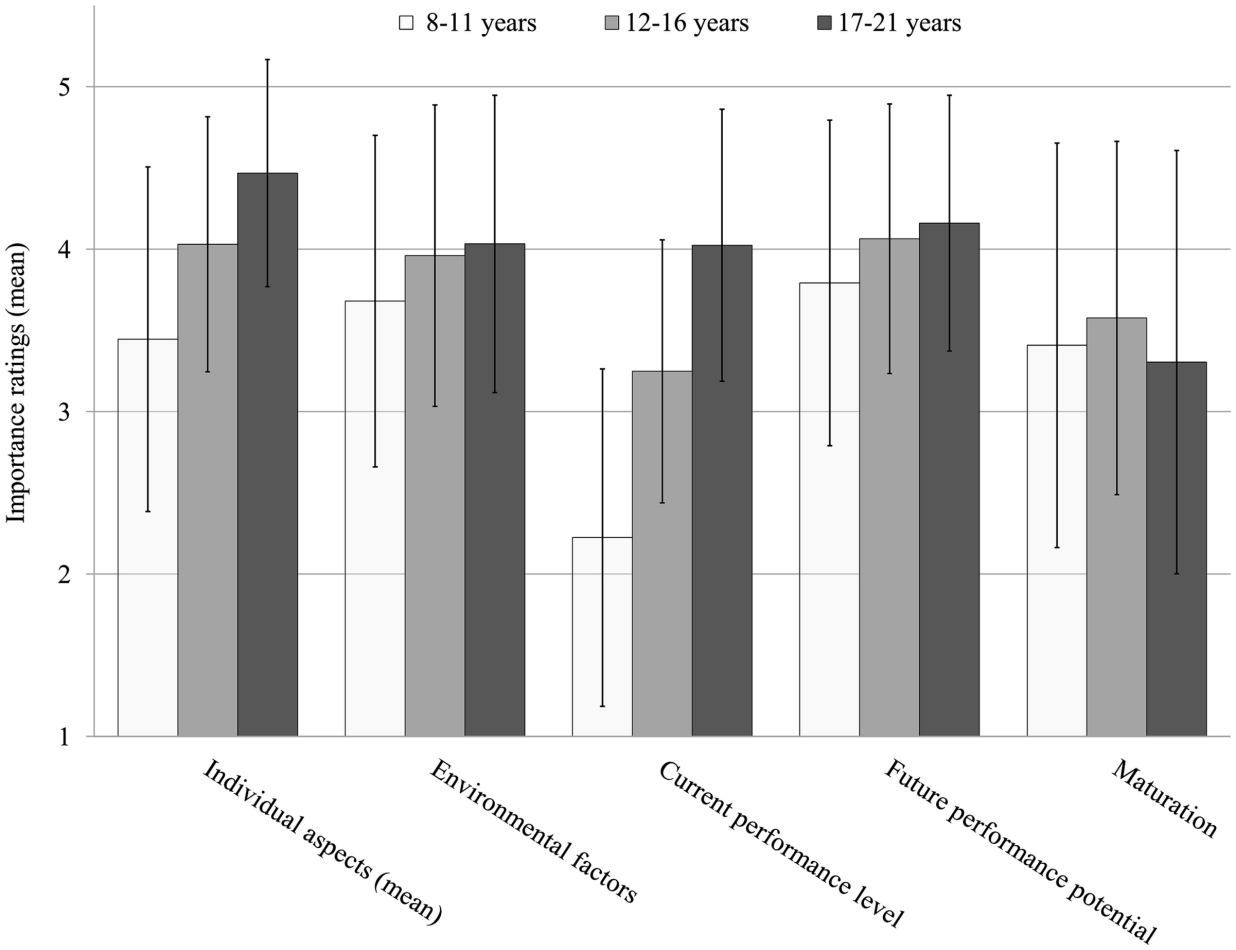
Coaches’ importance ratings for variables differentiated by age groups (whiskers show standard deviations).

Descriptively, results show that importance ratings of *individual aspects (mean)*, *environmental factors current performance level* and *future performance potential* increase with increasing player age and reach the highest importance ratings in the oldest age group. Variable *current performance level* starts with the lowest importance value (*M* = 2.22) and afterwards shows the biggest increases. The importance of *maturation* descriptively increases first before slightly decreasing afterwards (see Figure 2). All variables besides *maturation* show an importance rating of above 4 in the oldest age groups with individual aspects (mean) having the highest value (*M* = 4.47).

Inferential RM-ANOVA results showed statistically significant differences for all items but *maturation* (*p* = 0.102) with large effects for variables *individual aspects (mean)* and *current performance level* and medium effects for *future performance potential* and *environmental factors* (see Table 1). That is, the latter four variables showed statistically significant and practically relevant differences for the different age groups.

For variables *individual aspects (mean)* and *current performance level*, both contrasts (8–11 vs. 12–16 and 12–16 vs. 17–21) showed statistically significant and large increases in importance with increasing age (*p*s < 0.001, *η_p_^2^* = 0.43–0.67). That is, the effects’ practical relevance increases with player age.

For variables *environmental factors and future performance potential*, contrasts showed statistically significant increases with medium effects (*p*s < 0.001, *η_p_^2^* = 0.09 and 0.11, respectively) for contrasts 8–11 vs. 12–16, while contrasts 12–16 vs. 17–21 showed statistically non-significant and small effects (*p* = 0.382, *η_p_^2^* = 0.01 and *p* = 0.241, *η_p_^2^* = 0.01, respectively). That is, those two variables are most relevant in the younger and middle age group before staying rather equal in the oldest age group.

Variable *maturation* was the only variable to increase first and decrease afterwards, leading to statistically non-significant differences overall. However, both contrasts were statistically significant with small effects (*p* = 0.027, *η_p_^2^* = 0.04 and *p* = 0.035, *η_p_^2^* = 0.04, respectively). That is, maturation appears to be moderately important and relevant in all age groups with a small peak in age group 12–16.

## 4 Discussion

The aim of this study was to investigate basketball coaches’ general beliefs on defining and identifying talent as the basis for understanding the coach’s eye in basketball. In summary, findings suggest that coaches generally agree that talent can be identified to some degree and that they perceive it as a multidimensional, dynamic, and compensatory construct. These beliefs align with existing theoretical models of talent identification and development (e.g., Baker et al., 2019). Additionally, findings offer insight into the processes and assumptions underpinning the coach’s eye in athlete selection (in basketball). In the following, results are discussed not based on the two study parts but research themes.

### 4.1 Theme A: Talent is identifiable, multidimensional, and compensatory

Across the sample, coaches showed strong agreement with the statement that talent in basketball can be identified (75% of coaches rating 4 or 5). This aligns with prior research indicating that experienced coaches believe certain (performance) qualities, particularly those related to physicality, motor coordination, and basketball-specific skills, can serve as indicators of future performance potential (e.g., Rogers et al., 2022; Larkin et al., 2023). However, the overall pattern of ratings indicated a non-deterministic view of talent in basketball. Coaches appeared to acknowledge the probabilistic nature of talent identification and athlete selection, reflecting awareness that early performance does not always predict future success (Abbott and Collins, 2004; Till et al., 2016).

Coaches agreed with items reflecting a multidimensional view of talent in basketball (specifically items 2 and 3). This supports a growing body of literature that talent (in basketball) is not limited to technical or physical characteristics but also includes psychological, cognitive, and social–emotional domains (e.g., Baker et al., 2019; Johnston et al., 2018; Joseph et al., 2021). This strong agreement reflects a shift towards a holistic athlete evaluation, potentially reached based on coach’s eye evaluations. Additionally, the pattern of ratings indicated novel and broad support for the compensatory nature of talent in basketball (item 4). Many coaches agreed that weaknesses in one can be compensated for by strengths in another area (Vaeyens et al., 2008). However, while this compensation phenomenon is often cited and appears to play an important role, exact processes and potential combinations of certain qualities and how coaches can cognitively combine them is not well understood, especially in complex sports.

### 4.2 Theme B: Talent and its evaluation are dynamic

Present results show high levels of agreement with items indicating a dynamic (i.e., changing over time) idea of talent (in basketball). This supports approaches emphasizing longitudinal monitoring of athletes, as opposed to cross-sectional one-time assessments. Coaches appear to place substantial importance on incorporating an athlete’s development over time as they aim to base their selection decision on the future performance potential (peak performance age in basketball is often referred to as 25–30 years of age) rather than the current performance level (items 5–7). Based on the inferential contrast results, this effect appears to be even bigger at younger ages. Although coaches in the present study rate the importance of maturation on a rather constant medium level, players’ development and coaches’ selection behavior certainly are influenced by maturation differences, especially during puberty as also indicated by the importance peak in the age group 12–16 (e.g., Arede et al., 2021). The present results reflect coaches’ recognition of the non-linear and individualized nature of trajectories in sport that are crucial to young athletes’ development and thus selection decisions (e.g., Dunn et al., 2024; Baker et al., 2020). However, it is important to note that this differentiation may be challenging for coaches to implement in practice (Barraclough et al., 2024).

### 4.3 Coaches’ beliefs are dynamic too

Based on the ratings, coaches do not only see the athlete’s talents as dynamic, they also appear to recognize that their own beliefs and identification strategies evolve over time (item 8). These novel findings are in accordance with recent research (Roberts et al., 2025) and support the idea that the coach’s eye is not a fixed entity, but rather a dynamic, learned and adaptive mechanism shaped through professional learning and experience (Lath et al., 2021; Roberts, 2021; Christensen, 2009). Such findings have important implications for coach education programs as they should not only disseminate knowledge on athlete and talent identification frameworks but also foster critical reflection on coaches’ assumptions, biases, and decision-making processes when preparing and making those selection decisions.

### 4.4 Theme C: Importance of both individual and environmental factors

In the present study, coaches showed strong support for the important roles of both individual and environmental factors for athlete development and selection processes (items 9–11). For example, this includes anthropometric, tactical and technical aspects on the one hand, and access to coaching, family support, and training conditions on the other hand. Given the inferential contrast results, environmental factors appear to be most important in younger age groups. Coaches appear to include both information into their tasks of planning and designing training programs and selecting athletes as part of athlete development (Sargent Megicks et al., 2023). By acknowledging both individual aspects and environmental factors, coaches demonstrate an appreciation of the interactive nature of talent development (in basketball). This interaction is crucial for further progress in both science and practice, and is incorporated in more integrative models of youth sport (Dorsch et al., 2022). As environmental factors are often the prerequisite for individual development opportunities, clubs and federations should acknowledge the importance of environmental factors when distributing resources (e.g., infrastructural and personal) towards different teams and measures.

### 4.5 Theme D: Importance of both objective and subjective data

Coaches showed a tendency of stronger agreement with subjective evaluations based on their coach’s eye (mainly practice or competition observations) than with those emphasizing objective measurement tools (e.g., physical performance testing, statistics). This finding is consistent with previous research noting that coaches often rely on experiential knowledge and intuitive decision-making when assessing athletes and their talents (Lath et al., 2021; Roberts, 2021; Christensen, 2009). Objective tools are increasingly used within athlete evaluation and selection processes given advances in sports analytics. However, coaches appear to treat these as secondary or supportive rather than primary decision-making tools. This prioritization of subjective over objective data may be explained by the enormous complexity and context-dependence of evaluating an athlete in a given sports game, e.g., basketball. Current measurement tools often have limitations in capturing psychosocial and game-based qualities in highly representative contexts. Given the vulnerability of subjective evaluations for biases, integrating objective data as a validating or counterbalancing force remains a critical consideration in evidence-based coaching and athlete selection processes. Coaches should aim to have all objective results (e.g., anthropometrics, sprint times, game stats) present to incorporate those with their subjective evaluations through a social exchange process with other coaches. Despite often not explicitly tangible, this overall combination and weighting of factors (as a team of selection decision makers) appears crucial for the selection process. In summary, this appears to be the current status: Coaches make their selections decisions based on their coach’s eyes while incorporating more or less information from objective assessments. Future research must further investigate this crucial interplay as part of coach’s eye understanding.

### 4.6 Limitations

No research comes without limitations. The present study had a focus on (a) German coaches and their beliefs in the context of (b) male basketball players in 5v5 basketball. Although most findings should be transferable to other contexts, future research should include other populations (e.g., female) and 3×3 basketball. Furthermore, in hindsight, some items used in the questionnaire appear not perfectly concise in their wording. Future research should rephrase those items (e.g., change terms like “*as many* objective parameters […] *as possible*”) to ensure most valuable data. Also, a lack of longitudinal data and possible biases from self-report data acquisition may have influenced the findings. Lastly, rating the importance of objective and subjective data should have been included in the age differentiation part to gain further insight.

## 5 Conclusions and practical implications

All in all, the present study provides quantitative evidence that basketball coaches tend to view talent in basketball as a partly identifiable, yet fundamentally complex, multidimensional, and dynamic construct. With that, coaches’ beliefs and widespread research themes appear to overlap. Coaches value longitudinal development and assessment, they integrate individual and environmental factors, and prioritize subjective evaluations over objective assessments while also acknowledging their relevance. These beliefs highlight the continued importance of holistic, flexible, and developmental approaches to athlete evaluation. While objective tools and metrics appear to have a place in selection processes, coaches still rely heavily on experience-based interpretations of their own coach’s eye observations. This may both enrich and complicate athlete selection decisions, and findings should be transferable to other sports and contexts. Coaches within one program or system should work towards a consistent understanding of *how* (e.g., objective vs. subjective approaches) they look for *what* (e.g., individual vs. environmental factors) in different age groups and contexts. Finally, findings support the view that coaching expertise and its underpinning beliefs are themselves evolving, suggesting a need for reflective and evidence-informed coach development systems that can adapt to the nuanced realities of talent identification and athlete selection (in basketball).

## Data Availability

The datasets presented in this article are not readily available because of confidentiality. Requests to access the datasets should be directed to TK, till.koopmann@uol.de.
